# A Sparse Autoencoder and Softmax Regression Based Diagnosis Method for the Attachment on the Blades of Marine Current Turbine

**DOI:** 10.3390/s19040826

**Published:** 2019-02-17

**Authors:** Yilai Zheng, Tianzhen Wang, Bin Xin, Tao Xie, Yide Wang

**Affiliations:** 1 Department of Electrical Automation, Shanghai Maritime University, Shanghai 201306, China; zhengyleli@163.com (Y.Z.); xbbx385@163.com (B.X.); 201840210002@stu.shmtu.edu.cn (T.X.); 2 Institut d’Electronique et Telecommunications de Rennes (IETR), University of Nantes, 44306 Nantes, France; yide.wang@polytech.univ-nantes.fr or yide.wang@univ-nantes.fr

**Keywords:** marine current turbine, blade attachment, sparse autoencoder, softmax regression

## Abstract

The development and application of marine current energy are attracting more and more attention around the world. Due to the hardness of its working environment, it is important and difficult to study the fault diagnosis of a marine current generation system. In this paper, an underwater image is chosen as the fault-diagnosing signal, after different sensors are compared. This paper proposes a diagnosis method based on the sparse autoencoder (SA) and softmax regression (SR). The SA is used to extract the features and SR is used to classify them. Images are used to monitor whether the blade is attached by benthos and to determine its corresponding degree of attachment. Compared with other methods, the experiment results show that the proposed method can diagnose the blade attachment with higher accuracy.

## 1. Introduction

To date, reducing carbon emissions has become a consensus around the world. It is urgent to adjust the energy structure, reduce the dependence on fossil energy, and increase the use of sustainable energy, which makes the wind, solar, and marine current energies [1,2,3] more and more attractive. The system of wind and solar energies is greatly affected by the environment, which occupies a lot of land resources, and brings noise and visual pollution to surrounding residents. The marine current energy can avoid these problems. The marine current mainly refers to the steady flow in the submarine channel, and the regular flow of water caused by the tides [4]. The flow of the marine current is stable, and the flow rate is kept within a certain range all year round [5], therefore power can be continuously generated [6,7]. Marine current energy is an inexhaustible green energy resource and the marine current turbine (MCT) is mainly independent of weather conditions [8]. However, compared with the terrestrial environment, the undersea working environment is more complex. In addition to the traditional generator faults, the MCT system is also influenced by the marine environment, such as attachment, biofouling [9,10], etc., affecting the normal operation of the electrical equipment. On the other hand, the marine current generation system is affect by the sun, lunar gravity and the surge. The resulting instability of the current flow rate [11,12] makes the MCT work in a complicated environment for a long time, which means that the detection and diagnosis of the faults of the MCT are more difficult. The faults can cause great damage to the whole system, if not found and dealt with in time. The conventional faults caused by attachment include rotor asymmetries, increased surface roughness and the deformation of blade [13]. In addition, the metal parts are much easier corroded by attachment [8]. When sea creatures attach the blades, the blade imbalance and hydrodynamic will affect the results of the output power imbalance. The amplitude and frequency of the output voltage reduce while the blade is affected by the attachment. The attachment reduces the efficiency of the absorption of kinetic energy from the flow and reduces the rotational speed of the blades. At the same time, a small change of the flow rate has a greater influence on the output voltage [14]. If the blade attachment is not found in time and cleaned up immediately, the situation of biological deposition becomes serious and its output voltage waveform will be distorted.

At present, there is little research on the fault diagnosis of MCT. Reference [15] proposes a fault detection method, based on the empirical mode decomposition (EMD) and the spectral analysis for MCT under the conditions of waves and turbulence. A mode-correlation principal component analysis method is proposed to monitor MCT under the random occurrence of turbulence and waves [16]. Reference [17] uses the time domain, time-frequency domain and angle domain features to detect faults that achieve good performance for MCT under complex conditions. However, those methods only detect the imbalance fault. Particularly in reference [17], only two categories of faults (imbalance fault 1% and 3%) are considered, which means that the even-distributed attachment cannot be detected. Meanwhile, these methods still need humans to analyze the observed results. Reference [18] proposes a modified extended Kalman filter (MEKF) fault detection strategy, but this method needs an extra electric circuit, which is a challenge in an undersea environment.

On the other hand, electrical and mechanical signals are not always enough to diagnose faults in the environment with strong currents and complex spatiotemporal variability [19,20]. The undersea radio signals cannot travel far due to absorption losses [21] and many acoustic signals are lost due to partial band interference [21]. So, images of underwater camera are used as the fault-diagnosing signal in this paper. This provides an effective nondestructive means for underwater measurement in various scenarios [22]. In reference [23], a lithium polymer battery of 10,000 mAh capacity is used for the camera battery and the camera can work for up to 10 days, if it is controlled to record 60 seconds of video every two-hours under the sea with a depth between 1000 m and 1800 m. Traditional image classification methods include the BP neural network [24], support vector machine (SVM) [25], and principal component analysis [26], etc. The BP neural network and SVM require a great number of parameters when the dimension of the input is large. The CNN (Convolutional Neural Network), a more recent classification method, achieves high accuracy in image classification by stacking convolutional layers or blocks [27,28]. This also means a large number of parameters and very high computational complexity [29]. Some of the recent methods for image recognition are that the convolutional kernels and the softmax’s parameters, and the number of convolutional layers is greater than one. The mentioned method could extract abundant features by trained convolutional kernels for an image with complex features. However, a network with less convolutional layers also shows good performance in some image classifications. For instance, reference [30] uses two convolutional layers to classify different numbers. This paper tries to use one convolutional layer, and asynchronously trains convolutional kernels and softmax’s parameters. Convolutional kernels are trained by a sparse autoencoder (SA). A diagnosis method based on a sparse autoencoder and softmax regression (SR) is proposed to diagnose whether the blade of the MCT is attached by benthos and to determine its corresponding degree of attachment. Theoretical analysis and experimental results show the effectiveness of the proposed method.

This paper is an extended version of the method in reference [31] and the rest of paper is organized as follows. Section 2 introduces the problems of blade attachment. Section 3 describes the proposed method. Section 4 presents the platform and gives some experimental results and comparison. The conclusions are drawn in Section 5.

## 2. Problem Description on Blade Attachment of MCT

At present, MCT fault detection mainly focuses on imbalance faults, which are based on electrical signals. However, the electrical signal is affected by the complex environment, which results in difficulties to diagnose the attachment with similar degrees. In reference [17], two attachment degrees are set, which can be explicitly distinguished under waves, but cannot be distinguished under conditions of turbulence.

**Figure 1:**
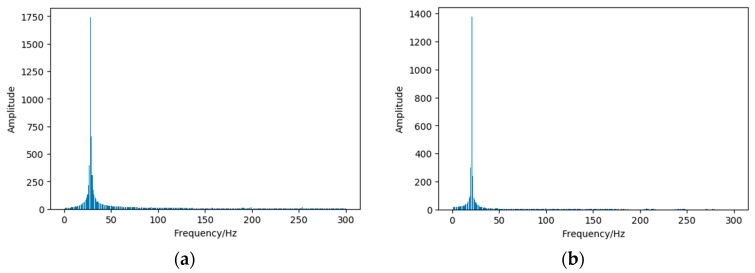
The output voltage of the marine current turbine (MCT) under different conditions: (**a**) The output voltage under a health condition; (**b**) The output voltage with uniform attachment.

The increased surface roughness and the deformation of the blade are also important, in addition to the rotor asymmetries caused by the imbalance attachment. These two kinds of faults are mainly caused by symmetrical or uniform attachment. For example, the output voltage signals are sampled under health conditions and uniform attachment; FFT (Fast Fourier Transformation) is used to analyze the sampled signal. The results are shown in Figure 1. Because it is difficult to distinguish between a health condition and uniform attachment condition for the amplitude and main frequency in the output voltage. This leads to the challenge of an accurate diagnosis based on the electrical signal under the increased surface roughness, and the deformation of blade. An acoustic signal is also used to diagnose faults under the increased surface roughness of the blade for the wind turbine [13]. However, many acoustic signals are lost in the undersea environment [21].

MCT’s image is used as the fault-diagnosing signal in this paper. The undersea environment is different from that on land, as there is no source of light. Underwater imaging systems have to rely on artificial light to provide illumination, which produces problems due to light absorption, light reflection, bending, light scattering and poor visibility [32]. Therefore, the image feature extraction method is a key point for diagnosing faults based on image classification.

**Figure 2:**
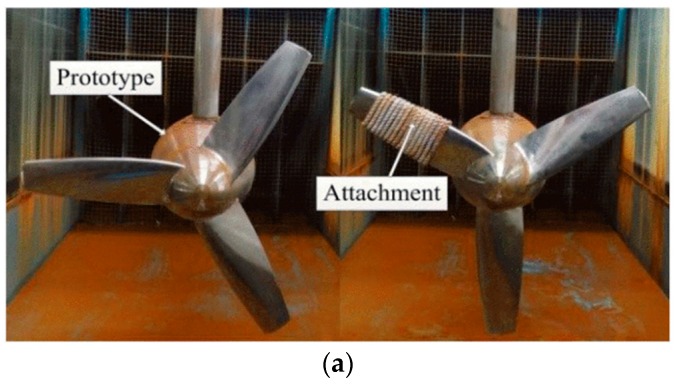

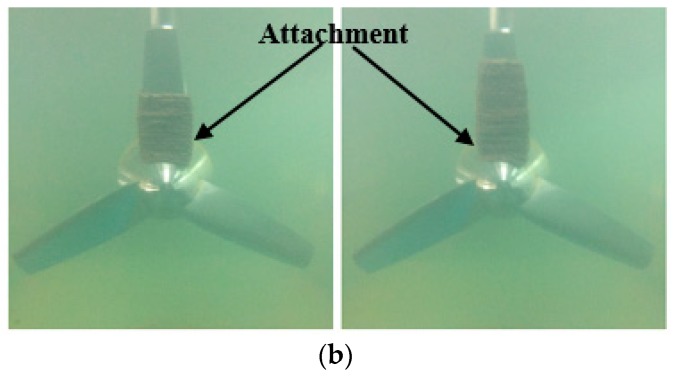
Image under different environments. (**a**) Waterborne image [17]; (**b**) Underwater image.

The MCT is salvaged from undersea with a thin attachment [8]. In addition, real biofilms were not able to be grown on a rotating turbine, or tested in the towing tank [33]. Blades were fouled with a 1.1 mm thick layer of lithium grease in reference [33]. Ropes used to simulate attachment in this paper are shown in Figure 2. Marine biofouling is a process from being attached to biological reproduction and takes about three-weeks [9]. By analyzing the images, and the degree of attachment, consequently, the degree of fault could be estimated in time. This kind of diagnosis method has been applied in cancer-image processing and has achieved promising results, such as the diagnosis of breast cancer [34].

## 3. The Sparse Autoencoder and Softmax Regression Based Diagnosis Method

**Figure 3:**
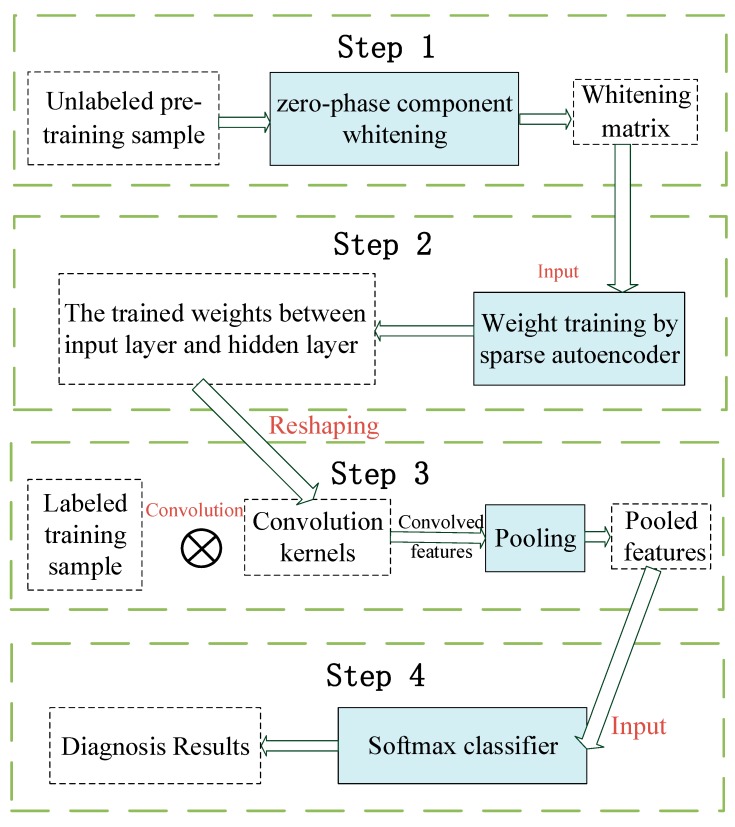
Frame of the proposed diagnosis method.

The diagnosis method proposed in this paper is divided into four steps as shown in Figure 3. Step 1, preprocessing the unlabeled images to pre-train the convolution kernels; Step 2, making the convolution between the labeled images and the convolution kernels to obtain the convolved features of each image in the labeled samples; Step 3, transforming the convolved features into the pooled features by using a pooling operation; and finally, Step 4, putting the pooled features into the softmax classifier to diagnose the faults category.

### 3.1. Image Data Preprocessing

The MCT images are used to extract patches for effectively extracting features. We extracted 500 patches of 20 × 20 pixels per channel (3 channels for each patch) from each image as the unlabeled learning samples, which are arranged in matrix $X_{unlabel}=\left[x_{unlabel}^{1},\ldots ,x_{unlabel}^{k},\ldots \right]$,where $x_{unlabel}^{k}$ is the *k*th column of $X_{unlabel}$. Then we used the zero mean and zero-phase component (ZCA) whitening technique [35] to calculate matrix $X_{whitening}$. The row images of MCT are effectively reduced by preprocessing of ZCA so as to sparse autoencoder’s input with low correlation.

(1) $$x_{unlabel}^{*k}=x_{unlabel}^{k}-\frac{1}{m}\overset{m}{\underset{i=1}{\sum }}x^{i}{}_{unlabel}$$

(2) $$C_{X}=\frac{1}{m}X_{unlabel}^{*}{\left(X_{unlabel}^{*}\right)}^{T}$$

(3) $$X_{whitening}=U{\left(S+I\right)}^{-\frac{1}{2}}X_{unlabel}^{*}$$

where $x_{unlabel}^{*k}$ is the kth column of $X_{unlabel}^{*}$; ***C_X_*** the covariance matrix of $X_{unlabel}^{*}$; *m* the number of samples; ***S*** is the eigenvalues of diagonal matrix and ***U*** is the eigenvectors of ***C_X_***, and ε is the regularization parameter.

### 3.2. Pre-Training Convolutional Kernels Based on Sparse Autoencoder

In classical CNN training, convolutional kernels and softmax’s parameters are simultaneously trained. In this paper, convolutional kernels are trained before training softmax’s parameters. Since the convolutional kernels and softmax’s parameters are trained asynchronously, SA is used to train the convolutional kernels.

**Figure 4:**
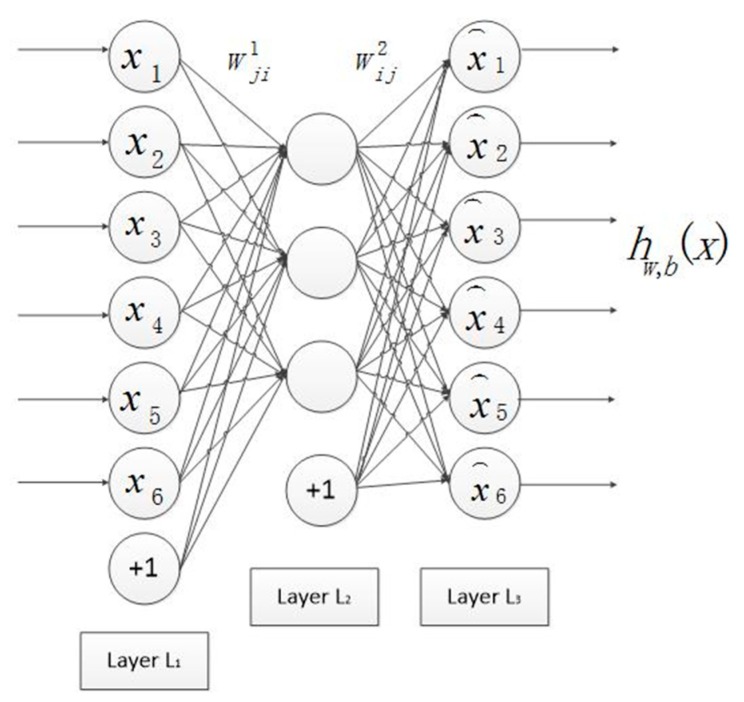
SA neural network structure [31].

Figure 4 shows the structure of the SA neural network. It has three layers: the input layer ($L_{1}$), the hidden layer ($L_{2}$) and the output layer ($L_{3}$), where “+1” is the bias coefficient. SA is an unsupervised learning algorithm because its ideal output equals to its input, which means that it can learn features from training data by itself. Assuming the preprocessed input matrix $X_{whitening}=\left[x^{1},\ldots ,x^{80000}\right]$, where $x^{k}$ is the *k*th column of $X_{whitening},\;x^{k}\in {\mathbb{R}}^{n}$, *n* = 1200 is the number of pixels of each patch. $W_{ji}^{\left(1\right)}$, for $i=1,\ldots ,s_{1},\;j=1,\ldots ,\;s_{2}$, denotes the weight connecting the *i*th neuron from the input layer to the *j*th neuron of the hidden layer. The input threshold of the hidden layer is $b^{\left(1\right)}$. $W_{ij}^{\left(2\right)}$, for $i=1,\ldots ,s_{3},\;j=1,\ldots ,\;s_{2}$, which denotes the weight connecting the *j*th neuron from the hidden layer to the *i*th neuron of the output layer; where $s_{1}=1200$ is the number of neurons in the input layer, $s_{2}=800$ the number of neurons in the hidden layer, $s_{3}=1200$ the number of neurons in the output layer. The threshold of the output layers $b^{\left(2\right)}$. $W_{ji}^{\left(1\right)}$, $W_{ij}^{\left(2\right)}$, $b^{\left(1\right)}$ and $b^{\left(2\right)}$ are trainable parameters, which are trained by the forward and backward propagation method. The activation function of the hidden layer is the sigmoid function and the output layer is the proportional function. The optimal values of parameters are calculated by using L-BFGS algorithm [36]. Finally, the weights of the hidden layer are the learned features. After pretraining based on SA, the weights between input layer and hidden layer are reshaped for extracting the convolution features as convolutional kernels.

(4) $$z_{j}^{\left(2\right)}=\overset{S_{1}}{\underset{i=1}{\sum }}W_{ji}^{\left(1\right)}x_{i}+b_{j}^{\left(1\right)}$$

(5) $$a_{j}^{\left(2\right)}=f_{1}\left(z_{j}^{\left(2\right)}\right)=\frac{1}{1+\exp\left(-z_{j}^{\left(2\right)}\right)}$$

(6) $$z_{i}^{\left(3\right)}=\overset{S_{2}}{\underset{j=1}{\sum }}W_{ij}^{\left(2\right)}a_{j}^{\left(2\right)}+b_{i}^{\left(2\right)}$$

(7) $$a_{i}^{\left(3\right)}=f_{2}\left(z_{i}^{\left(3\right)}\right)=tz_{i}^{\left(3\right)}$$

where, $x_{i}$ is the *i*th component of vector x, $z_{j}^{\left(2\right)}$ and $a_{j}^{\left(2\right)}$ correspond to the input and output of the activation function in the *j*th neurons of the hidden layer respectively, $z_{i}^{\left(3\right)}$ and $a_{i}^{\left(3\right)}$ correspond to the input and output of the activation function in the *i*th neuron of the output layer respectively, *t* is the proportionality coefficient.

### 3.3. Features Extraction Based on Convolution and Pooling

Local connection and weight sharing are the characteristics of the convolution layer, so using convolution can reduce the number of parameters and training complexity. In addition, the convolutional and pooling architecture can learn invariant features and reduce over-fitting [37]. Firstly, the convolved features will be extracted from each image, and then the pooled features will be obtained by aggregating the convolved features.

Different features activation value is obtained at each location in the image by convolving each image with the convolution kernels pre-trained in the previous step. Specifically, if the number of pixels of one image is $D_{image}\times D_{image}$ and the number of pixels of the convolution kernels is $D_{patch}\times D_{patch}$, the dimension of the convolved features is $\left(D_{image}-D_{patch}+1\right)\times \left(D_{image}-D_{patch}+1\right)$ [30]. Assuming the number of kernels for the hidden layer is equal to $n_{h}$, the dimension of a convolved feature is $\left(D_{image}-D_{patch}+1\right)\times \left(D_{image}-D_{patch}+1\right)\times n_{h}$.

The pooling operation is then introduced to reduce the dimension of the convolved features, while maintaining the invariant information and to improve the results of less over-fitting. Since the features of each category are not complex, the mean pooling is used in this paper [30]. 

### 3.4. Faults Classification Based on Softmax Classifier

After Step 3, the pooling features are obtained for the training classifier. According to the different attachment degrees, the different categories and labels are set. The pooling features are the input of softmax. Suppose θ is a parameter matrix, the L-BFGS iterative algorithm can be used to obtain parameter θ.

(8) $$h_{\theta }\left(x^{\left(i\right)}\right)=\left[\begin{array}{c} \begin{array}{c} p(y^{\left(i\right)}=1|x^{\left(i\right)};\theta )\\ p(y^{\left(i\right)}=2|x^{\left(i\right)};\theta ) \end{array}\\ \begin{array}{c} \vdots \\ p(y^{\left(i\right)}=k|x^{\left(i\right)};\theta ) \end{array} \end{array}\right]=\frac{1}{\sum_{j=1}^{k}e^{\theta_{j}x^{\left(i\right)}}}\left[\begin{array}{c} e^{\theta_{1}x^{\left(i\right)}}\\ e^{\theta_{2}x^{\left(i\right)}}\\ \begin{array}{c} \vdots \\ e^{\theta_{k}x^{\left(i\right)}} \end{array} \end{array}\right]$$

## 4. Experimental Analysis

### 4.1. Experimental Platform

**Figure 5:**
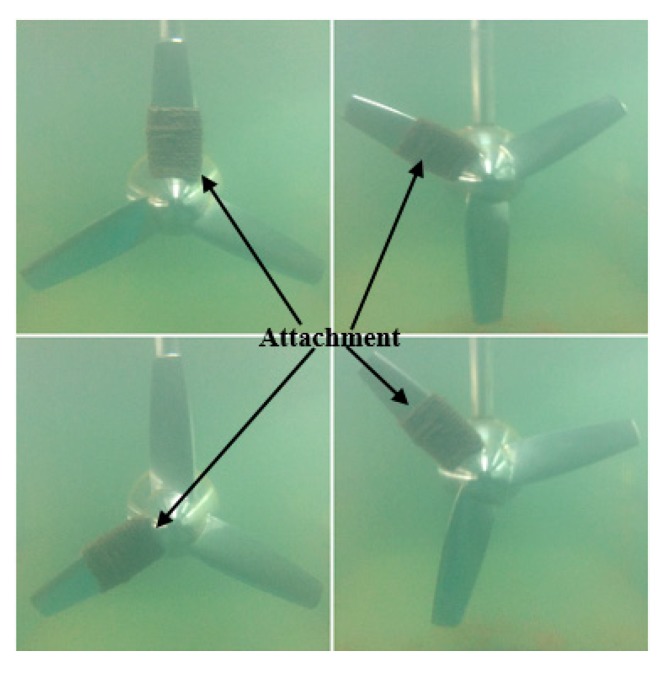
Four configurations of blade data acquisition.

In order to get a rich diversity of samples, the state of each category will be sampled from the blade in four different configurations to extract data, as shown in Figure 5. In this experiment, the speed of the water current is set to 0.6 m/s. 860 images with RGB channels collected by the underwater camera. The camera has 1.2 million pixels. The sampling frequency is 1 Hz. The luminous flux of fluorescent lamp is 1700 lm. After the remote transmission, each channel is represented by a matrix of size (320 × 320). Among them, 160 images are selected as unlabeled pre-training samples, 420 images as labeled training samples, and the remaining 280 images, as testing samples. The detail information is shown in Table 1 and Table 2.

**Figure 6:**
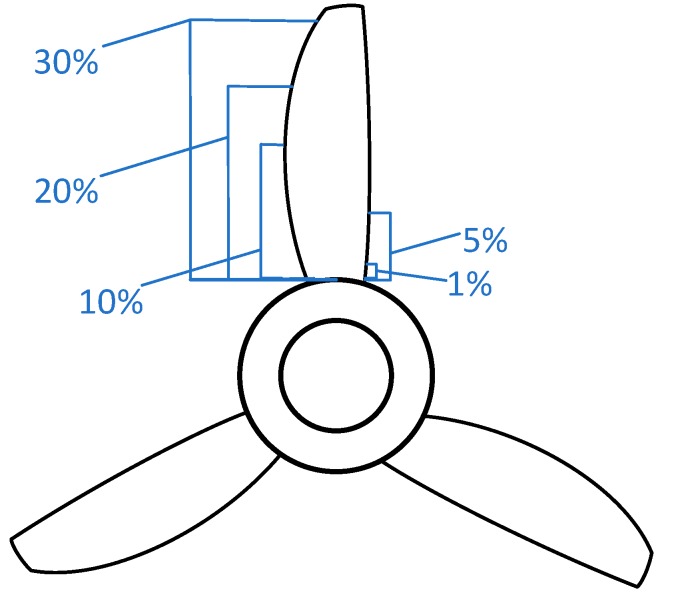
Single blade with different degrees attachment.

In this paper, for simplicity and without losing generality, we defined eight categories according to the proportion of the area covered by attachment, as shown in Figure 6. 

**Figure 7:**
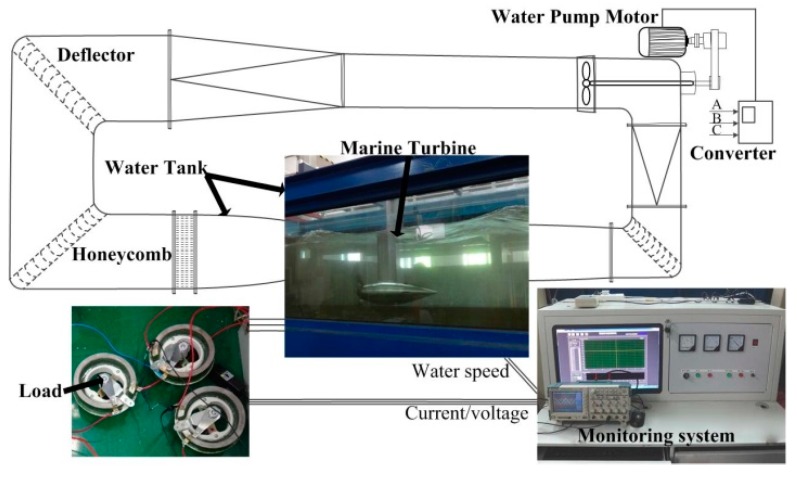
Experiment platform of the MCT [17].

Figure 7 shows the experiment platform of MCT, it is a 230 W direct-drive permanent magnet synchronous motor prototype. The whole system mainly consists of three parts: (1) the permanent magnet synchronous generator (PMSG) prototype; (2) the marine current simulation system (adjustable flow rate from 0.2 m/s to 1.5 m/s); (3) the data monitoring and collection system. This platform can simulate stationary current, waves and turbulence. Table 3 gives the main parameters of the system.

### 4.2. Experimental Results and Comparison

**Figure 8:**
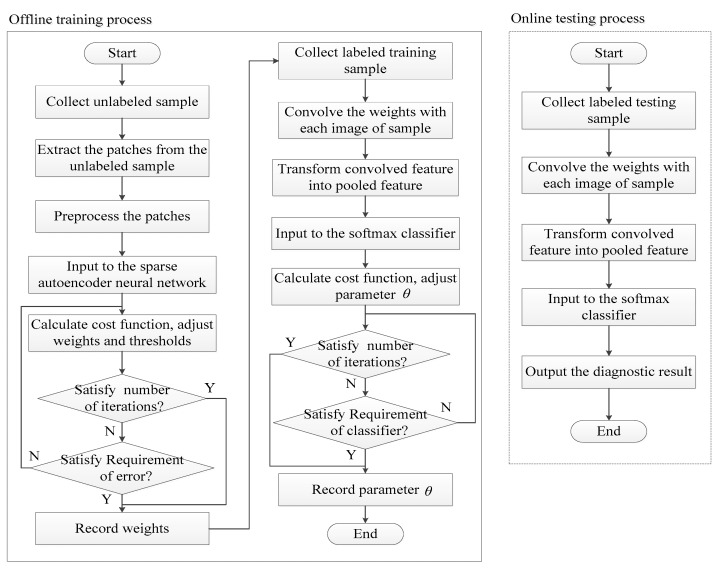
Training and testing flow chart.

Besides using the SA neural network and softmax classifier for features extracting and classifying, this paper also uses CNN for features of extraction and classification. The PCA (Principal Component Analysis) algorithm [38,39] for features extraction and BP neural network for classification [40], compares the results of different methods. The PCA algorithm is used to produce kernels from $X_{whitening}$ and the BP neural network is used to classify the faults, so the combination of the PCA algorithm with the BP neural network can produce kernels and classify faults, as seen in Table 4. Meanwhile, compared to CNN’s, the weights are different for this proposed method because the kernels and softmax’s parameters are simultaneously trained. Table 5 shows all the parameters of SA in training step and Figure 8 shows a flow chart for all of the steps. The parameters of the compared methods are shown in Table 4. It is just the training of the softmax’s parameters and convolution kernels of CNN that varies, the architecture of it is the same throughout.

Convolutional kernels and softmax’s parameters have been retrained. Meanwhile, white noise were added in the training sample and the test sample. The test has been repeated 20 times. Experimental results show that all the classifier method’s results are very good, under the same method of features extraction as shown in Table 6. That means the representational characteristics are obtained by the proposed method. As a result, the softmax classifier presents better performance than BP classifier. In addition, the softmax classifier shows a more stable diagnosis accuracy. The experimental results also show that the extraction ability of SA is better than that of PCA whatever its value of CPV (95% or 99%).

## 5. Conclusions

Due to the hardness of MCT’s working environment, underwater image is chosen as the fault diagnosing signal to classify the different degrees of MCT’s biological attachment. This paper proposes a diagnosis method based on a sparse autoencoder and softmax regression, which consists of four parts. (1) Preprocessing the unlabeled images to pre-train the convolution kernels; (2) making the convolution between the labeled images and convolution kernels to obtain the convolved features of each image in the labeled samples; (3) transforming the convolved features into the pooled features by using pooling operation; (4) putting the pooled features into the softmax classifier to diagnose the faults category. The SA is used to create kernels and the SR is used to classify them. Images are used to monitor whether the blade is attached by benthos and then to determine its corresponding degree of attachment. Also, this paper compares the simultaneous training method (CNN) with other asynchronous training methods (PCA for kernel production and BP for classification). The experimental results and comparison with other methods show that the proposed method is useful to classify the different degrees of biological attachment. The proposed method can also be applied to other fields [41,42,43,44,45,46]. However, the percentage of the area occupied by attachment is diagnosed in this paper. The types of attachment are not considered. In addition, the training time of the proposed method is too long. In the future work, we will think about the color and the thickness of the attachment, and we will simplify the algorithm to speed up the training time in the future research.

## Figures and Tables

**Table 1:** Diagnostic category label.

**Percentage of Area Occupied by Attachment (%)**	(0,1]	(1,5]	(5,10]	(10,20]	(20,30]	60 (two blades, with each 30 attachment)	90 (three blades, with each 30 attachment)
**Classifier Labels**	1	2	3	4	5	6	7

**Table 2:** Detail of dataset.

Dataset’s Name	Number
Unlabeled pre-training sample	160
Labeled training sample	420
Testing sample	280

**Table 3:** Parameters of the MCT.

PMSG	SAP 71
Rated power	230 W
Rated voltage	37 V
Rated current	21 A
Pole-pair number	8
Airfoil	Naca0018
Chord length	0.19 m–0.32 m
Blade diameter	0.6 m

**Table 4:** The parameters of mentioned methods.

Mentioned Methods	Parameters’ Name	Parameters
PCA	Cumulative percent variance	95% or 99%
BP (classifier)	Number of layers	2
	Loss function	Mean-square error
CNN	Number of convolutional layers	1
	Number of pooling layers	1
	Loss function	Cross entropy loss

**Table 5:** The parameters of the whole system.

Parameters	Significance	Value
ε	Whitening parameter	0.1
*m*	Number of training samples	80,000
$\lambda_{1}$	Weight attenuation parameter for SA	0.003
β	Weight of the sparsity penalty term	3
ρ	Sparsity parameter	0.1
$\lambda_{2}$	Weight attenuation parameter for softmax	0.0001
Hidden size	Number of neurons in the hidden layer	400
t	Proportionality coefficient	1

**Table 6:** Experimental results based on different methods.

Diagnosis Method		Average
PCA + BP	CPV = 95%	89.286%
	CPV = 99%	83.214%
PCA + softmax	CPV = 95%	93.929%
	CPV = 99%	96.429%
SA+BP		97.345%
SA+softmax		98.214%
CNN		97.500%

## References

[1] Muller N., Kouro S., Malinowski M., Rojas C.A., Jasinski M., Estay G. (2016). Medium-voltage power converter interface for multi-generator marine energy conversion systems. IEEE Trans. Ind. Electron..

[2] Ferreira R.M., Estefen S.F., Romeiser R. (2017). Under what conditions sar along-track interferometry is suitable for assessment of tidal energy resource. IEEE J. Sel. Top. App. Earth Observation Remote Sens..

[3] Lawrence J., Sedgwick J., Jeffrey H., Bryden I. (2013). An overview of the U.K. marine energy sector. Proc. IEEE.

[4] Zhou Z., Benbouzid M., Charpentier J.F., Scuiller F., Tang T. (2013). A review of energy storage technologies for marine current energy systems. Renew. Sustain. Energy Rev..

[5] Anwar M.B., Moursi M.S.E., Xiao W. (2016). Dispatching and frequency control strategies for marine current turbines based on doubly fed induction generator. IEEE Trans. Sustain. Energy.

[6] Goundar J.N., Ahmed M.R. (2014). Marine current energy resource assessment and design of a marine current turbine for Fiji. Renew. Energy.

[7] Chen H., At-Ahmed N., Machmoum M., Zam E.H. (2015). Modeling and vector control of marine current energy conversion system based on doubly salient permanent magnet generator. IEEE Trans. Sustain. Energy.

[8] Chen H., Tang T., Ait-Ahmed N., Benbouzid M.E.H., Machmoum M., Zaim E.H. (2018). Attraction, challenge and current status of marine current energy. IEEE Access.

[9] Cao S., Wang J.D., Chen H.S., Chen D.R. (2011). Progress of marine biofouling and antifouling technologies. Chin. Sci. Bull..

[10] Hsu H.H., Selvaganapathy P.R. (2014). Development of a low cost Hemin based dissolved oxygen sensor with anti-biofouling coating for water monitoring. IEEE Sens. J..

[11] Kavousi-Fard A., Su W. (2017). A combined prognostic model based on machine learning for tidal current prediction. IEEE Trans. Geosci. Remote Sen..

[12] Ren Z., Wang K., Li W., Jin L., Dai Y. (2017). Probabilistic power flow analysis of power systems incorporating tidal current generation. IEEE Trans. Sustain. Energy.

[13] Gao Z., Cecati C., Ding S.X. (2015). A survey of fault diagnosis and fault-tolerant techniques—Part I: Fault diagnosis with model-based and signal-based approaches. IEEE Trans. Ind. Electron..

[14] Zhou Z., Scuiller F., Charpentier J.F., Benbouzid M.E.H., Tang T. (2013). Power smoothing control in a grid-connected marine current turbine system for compensating swell effect. IEEE Trans. Sustain. Energy.

[15] Zhang M., Wang T., Tang T. (2017). An imbalance fault detection method based on data normalization and EMD for marine current turbines. ISA Trans..

[16] Zhou F., Park J.H., Liu Y., Wen C. (2016). Differential feature based hierarchical PCA fault detection method for dynamic fault. Neurocomputing.

[17] Zhang M., Tang T., Wang T. Multi-domain reference method for fault detection of marine current turbine. Proceedings of the 3rd Annual Conference of the IEEE Industrial Electronics Society (IECON 2017).

[18] Wang T., Liu L., Zhang J., Emmanuel S., Wang Y. (2019). A M-EKF fault detection strategy of insulation system for marine current turbine. Mech. Syst. Signal Process..

[19] Alvarez A., Caiti A., Onken R. (2004). Evolutionary path planning for autonomous underwater vehicles in a variable ocean. IEEE J. Oceanic Eng..

[20] Mcgee J., Catipovic J., Schoenecker S., Swaszek P. Interference suppression in congested undersea environments. Proceedings of the OCEANS 2015-Genova.

[21] Krishna C.R., Yadav P.S. A hybrid localization scheme for Underwater Wireless Sensor Networks. Proceedings of the International Conference on Issues and Challenges in Intelligent Computing Techniques.

[22] Huang L., Zhao X., Huang X., Liu Y. Underwater camera model and its use in calibration. Proceedings of the IEEE International Conference on Information and Automation.

[23] Cho H., Jeo H., Yu S.C., Lee J.K., Jeon M. Development of all-in-one-type deep-sea camera for monitoring Red Snow-crab habitats. Proceedings of the OCEANS 2016 MTS/IEEE Monterey.

[24] Xie J., Zhou J. (2017). Classification of urban building type from high spatial resolution remote sensing imagery using extended MRS and soft BP network. IEEE J. Sel. Top. App. Earth Observ. Remote Sens..

[25] Wang T., Qi J., Xu H. (2015). Fault diagnosis method based on FFT-RPCA-SVM for Cascaded-Multilevel Inverter. ISA Trans..

[26] Réjichi S., Chaabane F. Feature extraction using PCA for VHR satellite image time series spatio-temporal classification. Proceedings of the IEEE International Geoscience and Remote Sensing Symposium (IGARSS).

[27] He K., Zhang X., Ren S., Sun J. Deep residual learning for image recognition. Proceedings of the IEEE Conference on Computer Vision and Pattern Recognition (CVPR).

[28] Szegedy C., Liu W., Jia Y., Sermanet P., Reed S., Anguelov D. Going deeper with convolutions. Proceedings of the IEEE Conference on Computer Vision and Pattern Recognition (CVPR).

[29] Freeman I., Roese-Koerner L., Kummert A. Effnet: An efficient structure for convolutional neural networks. Proceedings of the 25th IEEE International Conference on Image Processing (ICIP).

[30] LÉcun Y., Bottou L., Bengio Y., Haffner P. (1998). Gradient-based learning applied to document recognition. Proc. IEEE.

[31] Xin B., Wang T., Tang T. A deep learning and softmax regression fault diagnosis method for multi-level converter. Proceedings of the IEEE 11th International Symposium on Diagnostics for Electrical Machines, Power Electronics and Drives (SDEMPED).

[32] Hou G., Pan Z., Huang B., Wang G., Luan X. (2018). Hue preserving-based approach for underwater colour image enhancement. IET Image Process..

[33] Walker J.M., Flack K.A., Lust E.E., Schultz M.P., Luznik L. (2014). Experimental and numerical studies of blade roughness and fouling on marine current turbine performance. Renew. Energy.

[34] Endre S., Michał Z., Eyras E. (2015). Detection of recurrent alternative splicing switches in tumor samples reveals novel signatures of cancer. Nucl. Acid. Res..

[35] Krsman V.D., Sarić A.T. (2017). Bad area detection and whitening transformation-based identification in three-phase distribution state estimation. IET Gen. Transm. Distrib..

[36] Ge F., Ju Y., Qi Z., Lin Y. (2018). Parameter estimation of a gaussian mixture model for wind power forecast error by Riemann l-bfgs optimization. IEEE Access.

[37] Norouzi M., Ranjbar M., Mori G. Stacks of convolutional Restricted Boltzmann Machines for shift-invariant feature learning. Proceedings of the 2009 IEEE Conference on Computer Vision and Pattern Recognition.

[38] Ge Z. (2018). Process data analytics via probabilistic latent variable models: A tutorial review. Ind. Eng. Chem. Res..

[39] Wang T., Xu H., Han J., Bouchikhi E.H.E., Benbouzid M. (2015). Cascaded h-bridge multilevel inverter system fault diagnosis using a PCA and multi-class relevance vector machine approach. IEEE Trans. Power Electron..

[40] Rad S.J.M., Tab F.A., Mollazade K. Classification of Rice Varieties Using Optimal Color and Texture Features and BP Neural Networks. Proceedings of the 7th Iranian Conference on Machine Vision and Image Processing.

[41] Wen C., Wang Z., Hu J., Liu Q., Alsaadi F.E. (2018). Recursive filtering for state-saturated systems with randomly occurring nonlinearities and missing measurements. Int. J. Robust Nonlinear Control..

[42] Wang Y., Ding F. (2017). A filtering based multi-innovation gradient estimation algorithm and performance analysis for nonlinear dynamical systems. IMA. App. Math..

[43] Xu X., Li S., Song X., Wen C., Xu D. (2016). The optimal design of industrial alarm systems based on evidence theory. Control Eng. Practice.

[44] Liu S., Ding F., Xu L., Hayat T. (2019). Hierarchical principle-based iterative parameter estimation algorithm for dual-frequency signals. Circ. Syst. Signal Process..

[45] Wen C., Wang Z., Liu Q., Alsaadi F.E. (2018). Recursive distributed filtering for a class of state-saturated systems with fading measurements and quantization effects. IEEE Trans. Syst. Man Cybern. Syst..

[46] Wan L., Ding F. (2019). Decomposition-and gradient-based iterative identification algorithms for multivariable systems using the multi-innovation theory. Circ. Syst. Signal Process..

